# Decoding the hypoxia-exosome-immune triad in OSA: PRCP/UCHL1/BTG2-driven metabolic dysregulation revealed by interpretable machine learning

**DOI:** 10.3389/fimmu.2025.1587522

**Published:** 2025-10-27

**Authors:** Weilong Ye, Yitian Yang, Feiju Chen, Xiaoxi Lin, Yunan Wang, Lianfang Du, Jingjing Pan, Weifeng Liao, Bainian Chen, Riken Chen, Weimin Yao

**Affiliations:** The Second Affiliated Hospital of Guangdong Medical University, Zhanjiang, Guangdong, China

**Keywords:** exosome signaling, obstructive sleep apnea (OSA), immune infiltration, machine learning, biomarkers

## Abstract

**Background:**

Obstructive sleep apnea (OSA) is a prevalent disorder characterized by significant metabolic and immune dysregulation. This study aims to uncover exosome-related biomarkers implicated in immune-metabolic disturbances in OSA and explore their potential as diagnostic and therapeutic targets.

**Methods:**

Transcriptomic data from two GEO datasets (GSE135917 and GSE38792) were integrated and analyzed using differential expression analysis via the *limma* package. Key biomarkers were identified using feature selection techniques including LASSO and Random Forest. Machine learning models, specifically XGBoost, were trained to evaluate biomarker performance, with model accuracy assessed by ROC curve analysis and AUC values. Immune cell infiltration was evaluated using single-sample Gene Set Enrichment Analysis (ssGSEA). Drug enrichment predictions were made through the Drug Signatures Database (DSigDB). Vivo and Vitro Experimental Validation on Multiple Independent cohorts.

**Results:**

Three exosome-related biomarkers—PRCP, UCHL1, and BTG2—were identified as central to OSA’s immune-metabolic dysregulation. XGBoost modeling demonstrated robust predictive power (AUC = 0.968). Immune analysis revealed significant correlations between gene expression and immune cell subsets, particularly CD56 bright natural killer cells and Memory B cells. Drug enrichment analysis identified potential therapeutic compounds, including Pentaphenate and Delphinidin, which target these biomarkers. OSA is associated with a reproducible transcriptional signature characterized by increased PRCP and UCHL1 expression and decreased BTG2 expression.

**Conclusions:**

This study identifies PRCP, UCHL1, and BTG2 as key exosome-related biomarkers in OSA that regulate immune-metabolic disruption. By integrating transcriptomic data, machine learning, and immune analysis, we uncover an “exosome-immune” axis in OSA pathophysiology.

## Graphical Abstract

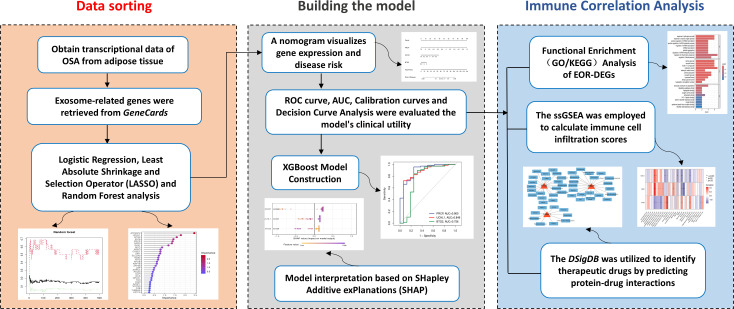

## 1 Introduction

An estimated 1 billion people worldwide are affected by OSA (1), and its prevalence continues to rise (2), primarily due to the increasing global rates of obesity (3–5). OSA is characterized by repeated partial or complete obstruction (collapse) of the upper airway during sleep, leading to hypercapnia, intermittent hypoxia (IH), and a reduction in blood oxygen saturation (2). The clinical diagnostic standard for OSA relies on polysomnography (PSG) (6). However, the high cost and time-intensive nature of this diagnostic method limit its application in the early screening and long-term treatment monitoring of OSA. As a result, identifying reliable biomarkers has become a research focus in the field of sleep medicine over the past decade (7). Pathophysiological studies suggest that IH, a core pathological feature of OSA, activates the sympathetic nervous system, induces metabolic disturbances, and promotes systemic inflammation and oxidative stress (8). Notably, IH exposure significantly upregulates the transcriptional activity of hypoxia-inducible factor-1α (HIF-1α), which then regulates a variety of downstream signaling pathways (9, 10). In terms of immune regulation, OSA patients exhibit characteristic proliferation of natural killer (NK) cells and natural killer T (NKT) cells (11). Further analysis shows that in mild to moderate cases, the proportion of CD4+ effector T cell subsets is abnormally elevated, while the numbers of effector memory T cells (TEM) and central memory T cells (TCM) are significantly reduced (12). Severe OSA cases display also pronounced immune dysregulation: the ratio of T helper (Th) cells to cytotoxic T lymphocytes (CTLs) decreases, while the number of B lymphocytes, which mediate humoral immunity, is significantly reduced (13). These findings suggest that the pathological progression of OSA involves complex immune cell dynamic imbalances, with characteristic immune phenotype changes observed at different stages of the disease. This provides potential targets for the development of novel diagnostic and therapeutic strategies.

Exosomes, key components of adipose-derived extracellular vesicles, play a crucial role in systemic metabolic regulation (14). These nanometer-sized vesicles, ranging from 30 to 150 nm in diameter, are rich in proteins and nucleic acids (including mRNA, miRNA, and lncRNA) derived from their parent cells (15). By mediating intercellular communication, metabolic waste clearance, and the maintenance of microenvironment homeostasis, exosomes significantly contribute to metabolic processes (16). Notably, exosome-carried metabolic regulatory factors can specifically bind to lipid transport proteins, modulating inflammatory cascades, immune response networks, and programmed cell death pathways (17, 18). This ultimately leads to pathological changes associated with metabolic disorders (19). Based on these functions, this study proposes an innovative hypothesis: intermittent hypoxia may alter the exosome secretion profile of adipose tissue, which in turn changes immune cell infiltration patterns, ultimately driving the pathological processes of OSA.

Current research has yet to fully elucidate the molecular mechanisms by which adipose-derived exosomes interact with metabolic regulation. Experimental evidence has shown that adipose tissue macrophages (ATMs) deliver miR-155 to adipocytes via exosomes, and this microRNA plays a significant role in improving obesity-related metabolic abnormalities by inhibiting the expression of peroxisome proliferator-activated receptor γ (PPARγ) (20). On the other hand, exosome-derived miR-34a from adipocytes has been shown to suppress M2 macrophage polarization, exacerbating the chronic inflammatory state induced by obesity (17). These findings suggest a bidirectional regulatory network between adipocytes and immune cells mediated by exosomes, offering a new perspective on the mechanistic study of metabolic diseases.

Building on this background, this study aims to adopt a comprehensive bioinformatics approach. First, it will screen OSA-specific exosome biomarkers and establish a machine learning-assisted diagnostic model. Second, the study will analyze the immune microenvironment of adipose tissue using the ssGSEA (single-sample Gene Set Enrichment Analysis) algorithm. Finally, we conducted *in vivo* and *in vitro* experimental validations across multiple independent cohorts and established a theoretical framework for the “hypoxia–exosome–immune” regulatory axis, thereby providing a solid foundation for the development of precise therapeutic targets.

## 2 Materials and methods

### 2.1 Collection and preprocessing of OSA transcriptomic data

The mRNA expression profiles for OSA were obtained from the GEO database, specifically datasets GSE135917 (21) and GSE38792 (22), both generated using the GPL6244 platform (Affymetrix Human Gene 1.0 ST Array). In the GSE135917 dataset, the control group included 8 samples, while the OSA group comprised 34 samples, with total RNA extracted from subcutaneous adipose tissue. Similarly, the GSE38792 dataset consisted of 8 control samples and 10 OSA patient samples, with RNA extracted from visceral adipose tissue biopsies collected during surgery. Log transformation was applied to both datasets to standardize expression values, followed by correction of distribution differences across samples. The datasets were then merged, and batch correction was performed to mitigate technical variations. Principal component analysis (PCA) was employed to visualize the differences between the two datasets before and after batch correction, ensuring improved data comparability.

### 2.2 Differential gene expression analysis and intersection with exosome-related genes

After data preprocessing, differential expression analysis was conducted using the *limma* package to compare gene expression profiles between control and disease groups, aiming to investigate the molecular mechanisms underlying sleep apnea. The *normalize-Between-Arrays()* function was applied to standardize the data. Subsequently, further analysis was performed using linear modeling: the *lmFit()* function was employed to fit a generalized linear model, *make-Contrasts()* was used to construct a contrast matrix defining specific comparisons, followed by *contrasts.fit()* for contrast analysis, and finally, *eBayes()* was applied for empirical Bayesian adjustment to enhance the robustness and accuracy of statistical inference. The filtering criteria included an adjusted p-value < 0.05 and |log2FC| > 0.5 (approximately corresponding to a 1.41-fold change). This threshold was chosen based on established practices in similar studies (23, 24), as microarray data typically reveal subtle expression changes, with a |log2FC| > 0.5 regarded as a meaningful difference. The resulting differentially expressed genes were visualized using a heatmap. Exosome-related genes were retrieved from the *GeneCards* database, a publicly available resource for human gene information (https://www.genecards.org/). We selected genes with Relevance Score > 2 as strongly associated genes, which accounted for more than 50 percent of the total. A Venn diagram was then constructed to visualize the intersection between exosome-related genes and differentially expressed genes, highlighting those with potential relevance to the study.

### 2.3 Functional enrichment analysis of EOR-DEGs

To explore the functional roles of exosome-related differentially expressed genes (EOR-DEGs), Gene Ontology (GO) and Kyoto Encyclopedia of Genes and Genomes (KEGG) pathway analyses were performed using the *clusterProfiler* package (25). Enrichment was considered significant when both p-values and adjusted p-values were less than 0.05. GO analysis encompassed 3 domains: biological processes (BP), cellular components (CC), and molecular functions (MF). The results of these enrichment analyses were visualized using bar-plots to highlight significant pathways and cnet-plots to illustrate the relationships between genes and their associated terms.

### 2.4 Logistic regression analysis and feature selection of EOR-DEGs

To assess the prognostic and diagnostic value of EOR-DEGs, univariate logistic regression was first applied, with the odds ratio (OR) and p-value used to identify genes significantly associated with prognosis and diagnosis (p < 0.05). Genes meeting this threshold were then subjected to feature selection using Least Absolute Shrinkage and Selection Operator (LASSO) regression (λ. min) and Random Forest (RF) analysis (Importance > 4) (26). The overlap of selected genes from both methods was visualized using a Venn diagram, identifying a set of key biomarkers for further clinical and mechanistic analysis. Subsequently, box plot was used to illustrate the expression levels of feature genes across different groups, and correlation plot was employed to visualize their interrelationships.

### 2.5 Construction and evaluation of a diagnostic model

A nomogram was developed to visualize the relationship between feature gene expression and disease risk, with coefficients derived from multivariate logistic regression. The model’s performance was evaluated using the Receiver Operating Characteristic (ROC) curve, with the area under the curve (AUC) indicating predictive accuracy. Calibration curves were constructed to assess the agreement between predicted and observed outcomes, while Decision Curve Analysis (DCA) evaluated the model’s clinical utility by assessing net benefit at various threshold probabilities.

### 2.6 XGBoost model construction

The XGBoost algorithm (27) was selected for its efficiency and robust performance in binary classification tasks. The feature genes were set as the predictors, with occurrence of OSA acting as response variable. The model’s predictive performance was evaluated using ROC curves. To minimize overfitting, 5-fold cross-validation was performed during model validation, alongside a reduced learning rate and limited maximum depth.

### 2.7 Model interpretation based on SHAP

We calculated SHAP (SHapley Additive exPlanations) (28) values to interpret the XGBoost model. The SHAP summary plot visualized their relative importance. Dependency plots were generated to illustrate the relationship between gene expression levels and disease risk. Additionally, SHAP force plots were used to analyze individual patient predictions, offering detailed insights into the gene-specific contributions to the probability of OSA occurrence.

### 2.8 Immune correlation analysis

The ssGSEA was employed to calculate immune cell infiltration scores, which were subsequently correlated with the expression of feature genes. Spearman’s correlation method was used to assess the relationship between immune cell activity and gene expression, with statistical significance determined for each correlation. The results were visualized in a heatmap, where the strength and significance of the correlations were clearly represented. The 28 immune cell–related gene sets were obtained from previously published studies (29, 30).

### 2.9 Drug enrichment analysis

The Drug Signatures Database (DSigDB) was utilized to identify potential therapeutic drugs by predicting protein-drug interactions. The DSigDB online platform (https://dsigdb.tanlab.org/), a publicly accessible database that integrates drug-associated gene expression data, was employed to explore drug-gene relationships, mechanisms of drug action, and opportunities for drug repurposing (31). Candidate drugs were identified by comparing the database’s drug gene expression signatures with disease-related gene expression profiles. The results were visualized using Cytoscape software (https://cytoscape.org/).

### 2.10 Vivo and vitro experimental validation

Human SW872 liposarcoma cells (n=6) and murine 3T3-L1 preadipocytes (n=6) were cultured under standard conditions, with 3T3-L1 cells induced to differentiate into mature adipocytes using a commercial induction kit. Male C57BL/6J mice (8 weeks of age; n=10) were randomly assigned to normoxia or chronic CIH exposure. This experiment was reviewed and approved by the Animal Welfare and Ethics Committee under review number: IACUC-20250701-299. Cells and mice were exposed to intermittent hypoxia (IH/CIH) with cyclic oxygen fluctuations, while controls were maintained under normoxia. Total RNA was extracted from cells and mouse adipose tissues, reverse-transcribed into cDNA, and analyzed by SYBR Green-based qRT-PCR. Gene expression was quantified using the 2^(-ΔΔCT) method with GAPDH as the internal control, and all reactions were performed in triplicate to ensure reliability. IHC was performed on FFPE iWAT sections using antibodies against PRCP, BTG2, and UCHL1, with DAB visualization, and staining was quantified as percentage positive area using ImageJ. The detailed methodological section has been added in the supplementary file. A brief overview of the process is presented in the **Graphical Abstract**.

### 2.11 Statistical analysis

The entire analysis was conducted using R software (version 4.4.2). During data collection, the *GEOmirror* and *idmap2* packages facilitated data retrieval and annotation. The *limma* and *sva* packages were utilized for dataset organization, correction, merging, and differential expression analysis. To ensure uniform distribution of expression values across all samples, quantile normalization was applied using the *normalize-Between-Arrays* function. After merging the datasets, the *ComBat* method was employed to correct for batch effects. For visualization, box plots were generated, and the Wilcoxon rank-sum test was applied for group comparisons. Correlation analysis was performed using Pearson’s correlation coefficient to assess the relationships between gene expression levels. Data are presented as mean ± SEM; qPCR was analyzed using per-sample ΔCt values and reported as 2^−ΔΔCt, while IHC results were quantified as the percentage of positive area (area%) per sample across predefined fields, with two groups compared using unpaired two-tailed t-tests, multiple groups analyzed by one-way ANOVA with appropriate *post-hoc* tests, and non-parametric alternatives applied when assumptions of normality or homoscedasticity were not met; P < 0.05 was considered statistically significant.

## 3 Results

### 3.1 Data integration and differential expression analysis

**Figure 1:**
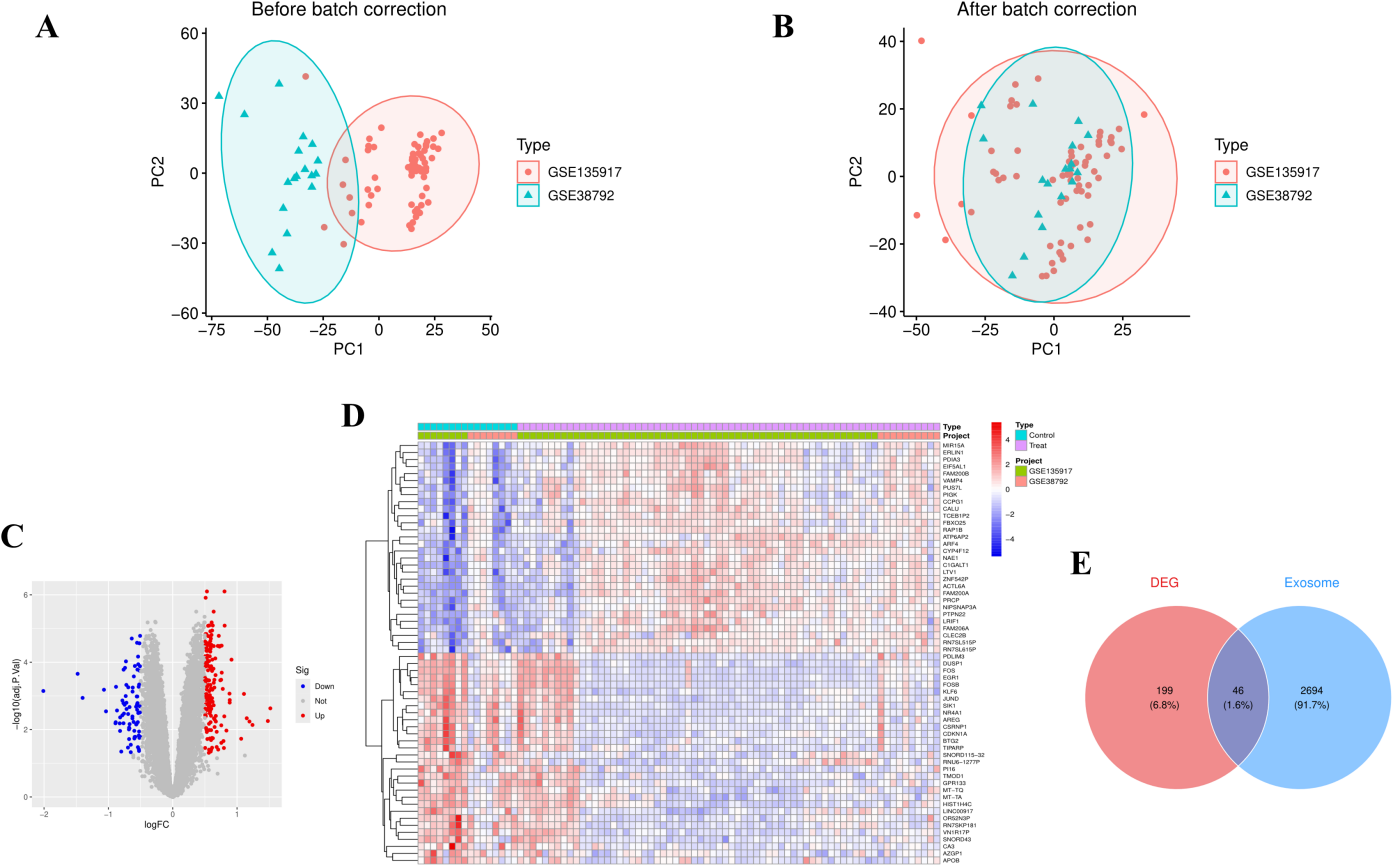
Data preprocessing and differential gene screening. **(A)** PCA plot before batch correction showed clustering by dataset origin. **(B)** PCA plot after batch correction, demonstrating clustering by disease status, indicating the removal of technical variations. **(C)** Volcano plot of differentially expressed genes (red dots: upregulated genes; blue dots: downregulated genes; thresholds: p-value < 0.05 and |log2FC| > 0.5), identifying 245 differentially expressed genes (DEGs). **(D)** Heatmap displaying the top 50 DEGs (ranked by |log2FC|), with row-normalized expression values (Z-score) reflecting expression patterns between OSA and control groups. **(E)** Venn diagram showing 46 exosome-related differentially expressed genes (EOR-DEGs, intersection of Gene Cards exosome gene set and DEGs).

PCA demonstrated that batch correction effectively mitigated batch effects, thereby preserving the integrity of the biological signal (**Figures 1A, B**). Addressing batch effects is crucial to minimize non-biological variations that could otherwise compromise the reliability of downstream analyses. Utilizing thresholds of p-value < 0.05 and |log2FC| > 0.5, 245 differentially expressed genes were identified (**Figure 1C**). A heatmap showcasing the top 50 upregulated or downregulated genes, ranked by |log2FC|, provides a visual representation of the key expression changes (**Figure 1D**). Using the keyword “exosome,” 5,293 protein-coding genes were identified, of which 2,740 genes with a Relevance Score > 2 were selected for further analysis. The Venn diagram displayed 46 EOR-DEGs (**Figure 1E**).

### 3.2 Functional enrichment analysis for EOR-DEGs

**Figure 2:**
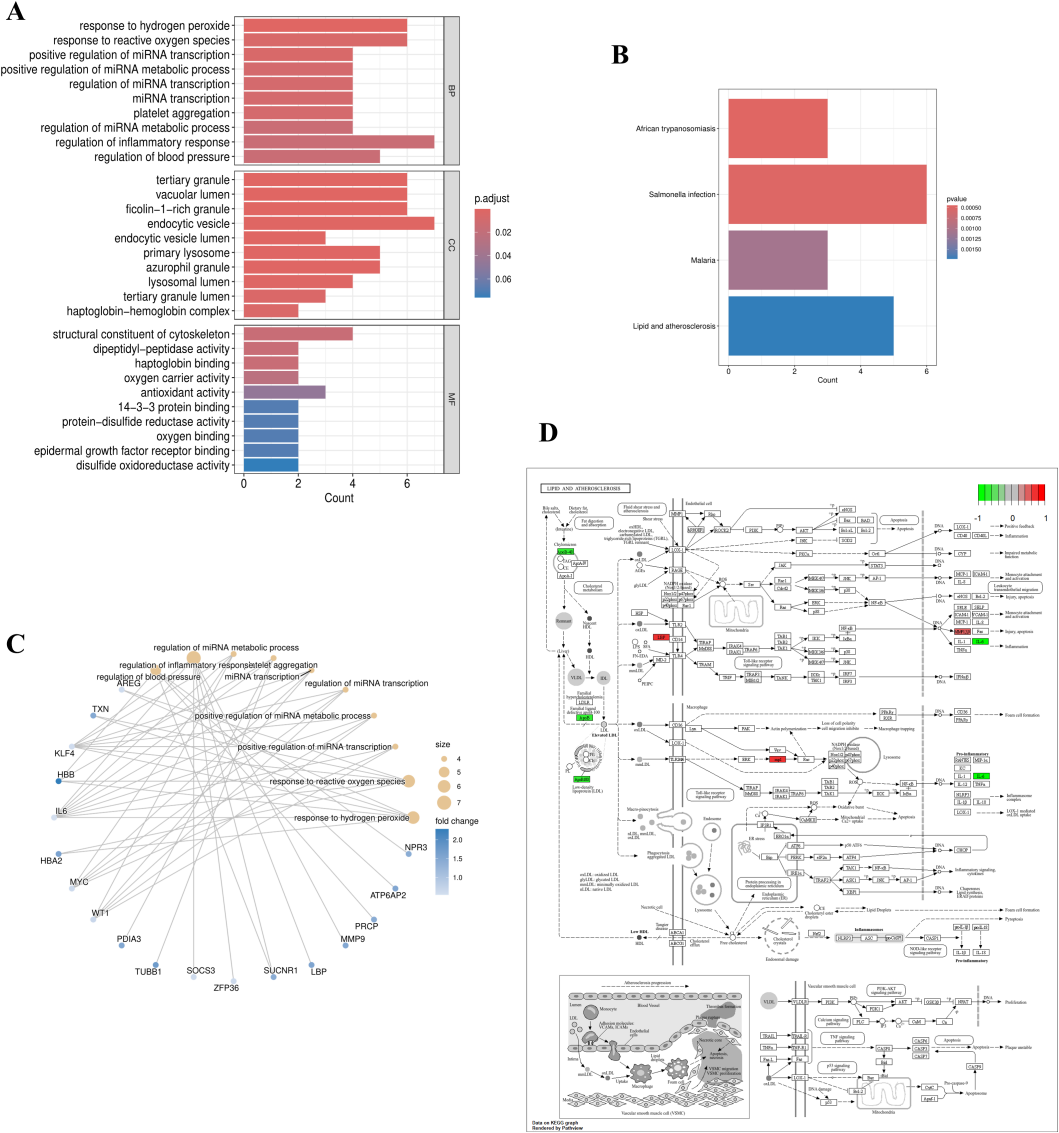
Functional and pathway enrichment of EOR-DEGs. **(A)** GO enrichment bar plot with significant terms (p < 0.05) including “regulation of inflammatory response” (BP), “endocytic vesicle” (CC), and “structural constituent of cytoskeleton” (MF). **(B)** KEGG pathway enrichment highlighted significant pathways such as “lipid metabolism” and “atherosclerosis”. **(C)** GO network diagram displaying the top 10 enriched terms, where node size represents the number of genes and edge width indicates gene overlap. **(D)** KEGG pathway map (lipid metabolism) highlighting key genes (LBP, MMP9, APOB, IL6, RAP1B).

The GO terms with the highest number of enriched genes in BP, CC, and MF were: regulation of inflammatory response, endocytic vesicle, and structural constituent of the cytoskeleton (**Figure 2A**). In the KEGG analysis, relatively few pathways were enriched (with a p-value < 0.05 and an adjusted p-value < 0.05 with a primary focus on lipid metabolism and atherosclerosis pathways (**Figure 2B**). The GO analysis network plot highlights the top 10 most significant functional enrichment categories (**Figure 2C**). The KEGG path view suggests that LBP, MMP9, APOB, IL6, and RAP1B are involved in lipid metabolism and atherosclerosis (**Figure 2D**).

### 3.3 Logistic regression analysis and feature selection of EOR-DEGs

**Figure 3:**
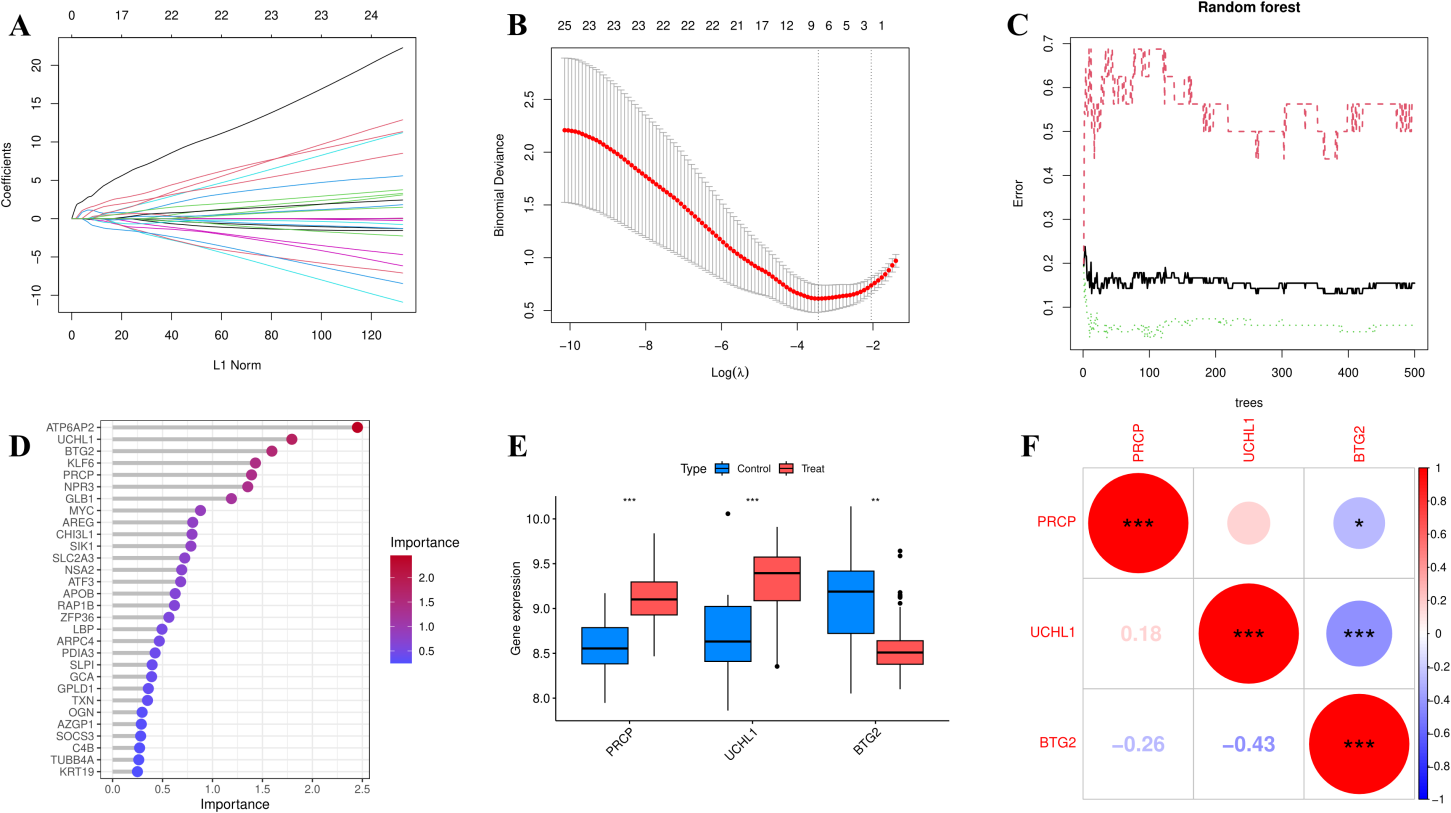
Feature selection was performed using LASSO (10-fold CV, lambda. min) and random forest (optimal trees, Mean Decrease Gini > 4). **(A)** coefficient path (lasso). **(B)** cross-validation error (lasso). **(C)** error rate curve (RF). **(D)** variable importance ranking (RF). **(E)** Boxplot showing the expression differences of feature genes (PRCP, UCHL1, BTG2) between the OSA group and the control group (*p<0.05, **p<0.01, ***p<0.001). **(F)** Heatmap of feature gene expression correlations (Pearson correlation coefficient).

In univariate logistic regression analysis, all 46 EOR-DEGs had p-values less than 0.05. Among them, 20 genes had odds ratios (ORs) less than 1, while the remaining genes showed ORs greater than 1 (**Table 1**). These 46 EOR-DEGs were further included in LASSO analysis to address potential collinearity, resulting in the selection of 10 genes (**Figures 3A, B**). Random forest analysis was employed to determine gene importance, with genes having importance scores greater than 4 being highlighted (**Figures 3C, D**). The intersection of genes identified through LASSO and RF analyses revealed three feature genes for model construction: PRCP, UCHL1, and BTG2. The box plot indicated that PRCP and UCHL1 were highly expressed in the OSA group, while BTG2 showed lower expression (**Figure 3E**). Correlation analysis revealed that UCHL1 was negatively correlated with both BTG2 and PRCP (**Figure 3F**).

**Table 1:** Univariate logistic regression analysis.

Gene	OR	OR.95L	OR.95H	p-value
PRCP	986.0791	60.17411	43735.75	2.90E-05
ATP6AP2	114.3371	14.29222	1758.272	8.39E-05
PDIA3	75.6924	11.11494	951.5929	0.000113
ARF4	47.53213	8.308052	428.4111	9.42E-05
GLB1	38.61957	7.375862	314.5474	0.000108
EXOSC3	27.94962	5.829075	190.7486	0.000158
UCHL1	27.675	6.2062	175.6567	7.80E-05
RAP1B	22.04958	4.769966	176.006	0.000579
ARPC4	15.57668	4.089505	76.78485	0.000201
TUBB4A	11.25975	3.354366	44.70034	0.000205
GCA	10.92942	3.585175	44.0934	0.000151
NSA2	8.126889	2.39504	39.09922	0.003046
SUCNR1	6.522213	2.137218	24.80007	0.002313
ALCAM	6.172922	2.180943	21.61758	0.001649
TUBB1	5.6868	2.185966	17.86299	0.001091
GPLD1	5.651804	2.053642	18.85715	0.00191
TXN	5.265804	1.945368	18.08795	0.003045
LBP	5.037635	1.938829	15.97191	0.002282
GPC4	4.393301	1.675899	13.27509	0.004583
HLA-DRB5	3.279293	1.375657	10.54385	0.019915
LYZ	2.892675	1.350791	6.975785	0.01005
CHI3L1	2.632013	1.409485	5.399355	0.004322
NPR3	2.523628	1.203417	5.78103	0.018065
MMP9	2.396216	1.199165	5.348592	0.020294
HBA2	2.236278	1.21276	4.4752	0.014438
HBB	2.092857	1.330592	3.531524	0.002651
ITLN1	0.62736	0.378592	0.949145	0.039053
C4B	0.477884	0.214379	0.907881	0.036145
IL6	0.477017	0.278475	0.779596	0.004177
SLPI	0.432136	0.19901	0.824733	0.017656
SLC2A3	0.392045	0.206861	0.702392	0.002305
KLF4	0.365831	0.186386	0.678436	0.00198
SOCS3	0.319353	0.147249	0.636311	0.001906
ZFP36	0.31906	0.148147	0.64201	0.001953
OGN	0.303189	0.124932	0.647185	0.003842
AREG	0.292776	0.124983	0.604413	0.001861
ATF3	0.283908	0.127676	0.582859	0.000993
MYC	0.270612	0.108965	0.590503	0.002069
WT1	0.259777	0.092083	0.626412	0.005114
DPP4	0.245084	0.073703	0.637639	0.009457
KRT19	0.215046	0.059646	0.574421	0.007162
APOB	0.158699	0.050603	0.423645	0.000567
AZGP1	0.138617	0.036829	0.396253	0.000937
SIK1	0.099512	0.026518	0.308981	0.000188
BTG2	0.068947	0.014537	0.254142	0.000202
KLF6	0.050211	0.009535	0.202541	9.92E-05

### 3.4 Construction and evaluation of a diagnostic model

**Figure 4:**
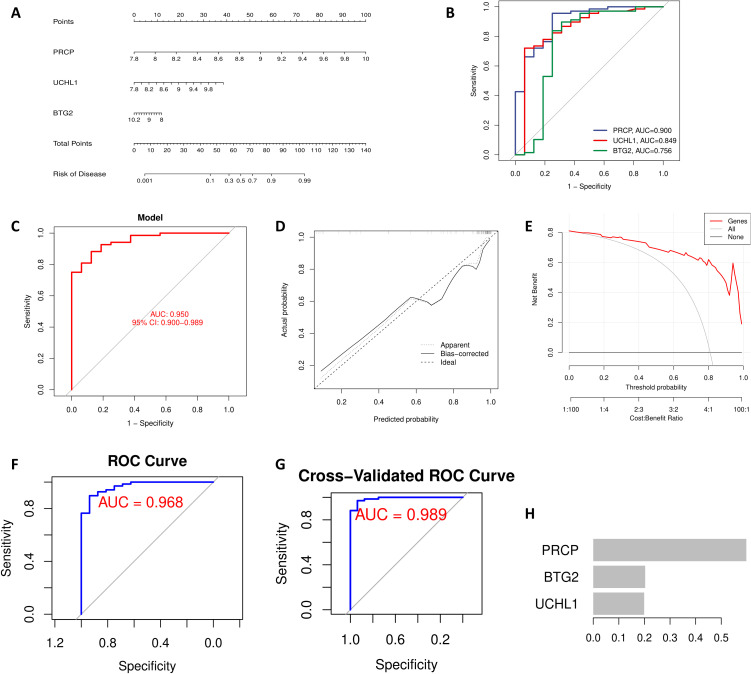
Feature gene-based OSA diagnostic model. **(A)** The nomogram integrates the expression levels of PRCP, UCHL1, and BTG2 to predict OSA risk, with the total score corresponding to the right-side risk axis. **(B)** ROC curve showing the performance of individual genes in predicting OSA. **(C)** ROC curve showing the performance of the combined diagnostic model based on feature genes. **(D)** Calibration curve with Bootstrap = 1000 iterations. The dashed line represents the ideal fit, and the solid line represents the model’s bias-corrected prediction. **(E)** DCA showing the net clinical benefit of the model when the threshold probability exceeds 10%. XGBoost Model Validation: **(F)** ROC curve. **(G)** 5-fold cross-validation. **(H)** Feature importance plot.

The nomogram visually represents a diagnostic model constructed through multivariate logistic regression analysis, leveraging the expression levels of hub genes to predict the risk of OSA (**Figure 4A**). The ROC curve (Bootstrapping method) demonstrates the model’s superior diagnostic performance, with an AUC value exceeding that of individual genes, confirming its robustness (**Figures 4B, C**). Model evaluation through the calibration curve indicates that the bias-corrected curve closely parallels the ideal curve, with only minor deviations observed in the high-probability range (approaching 1.0) (**Figure 4D**). Additionally, the Decision Curve Analysis (DCA) reveals that employing the model for prediction and intervention provides a higher net benefit (**Figure 4E**). These findings underscore the model’s reliability and practical utility in diagnostic applications. In the XGBoost model, the AUC value reached 0.968 (**Figure 4F**). To mitigate overfitting, 5-fold cross-validation was performed, further validating the model’s robustness (AUC = 0.989) (**Figure 4G**). Additionally, a feature importance plot was generated to illustrate the contribution of each gene to the model’s predictions (**Figure 4H**). For the XGBoost model, we set the following hyperparameters: learning rate (eta) = 0.01, maximum tree depth = 2, minimum child weight = 2, gamma = 0.1, subsample = 0.8, colsample_bytree = 0.8, lambda = 1, and alpha = 0. The objective was to use a smaller learning rate and limit model complexity to prevent overfitting when analyzing relatively small sample sizes.

### 3.5 Interpreting the machine learning model with SHAP analysis

**Figure 5:**
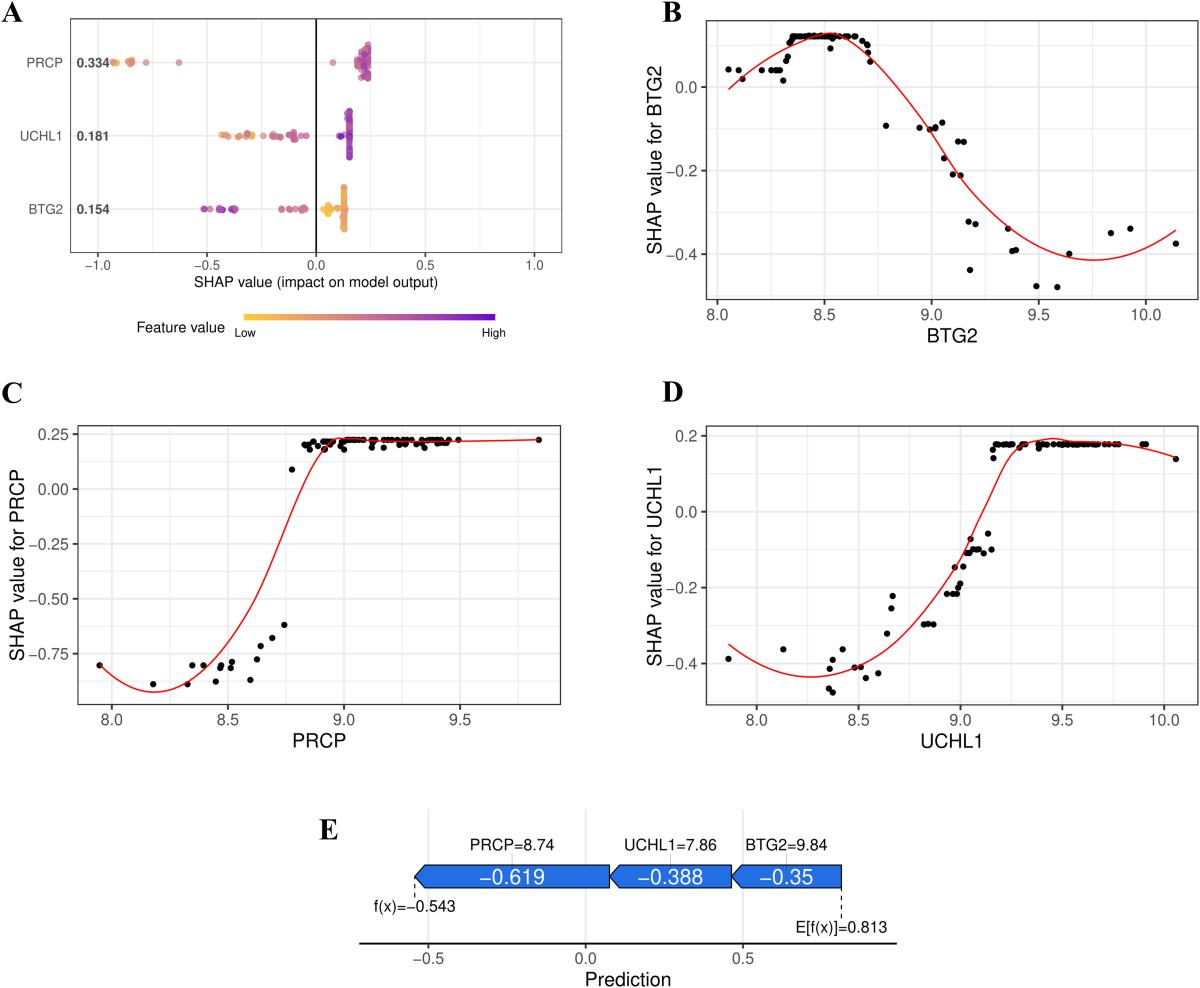
SHAP Interpretation of the XGBoost Model. **(A)** Summary plot illustrating the contributions of PRCP, UCHL1, and BTG2 to the overall prediction of OSA risk. **(B)** Relationship between BTG2 Expression Level and SHAP Value (LOESS fitting curve). **(C)** Relationship between PRCP Expression Level and SHAP Value. **(D)** Relationship between UCHL1 Expression Level and SHAP Value. **(E)** SHAP force plot illustrating the contribution of feature genes to the OSA risk prediction for a control sample.

The SHAP Summary Plot illustrates the contributions of the 3 hub genes (PRCP, UCHL1, and BTG2) to the overall model prediction (**Figure 5A**). Among them, PRCP shows the highest average SHAP value (0.334), indicating its strongest influence on the model’s predictions, while UCHL1 and BTG2 have relatively smaller contributions. The Dependence Plot visualizes the relationship between the expression levels of the feature genes and their corresponding SHAP values. For instance, the SHAP value for BTG2 peaks around an expression level of 8.5, decreasing as the expression level increases, suggesting a nonlinear relationship that may reflect BTG2’s complex regulatory role in the model’s output (**Figure 5B**). The figure also shows the relationships for PRCP and UCHL1 (**Figures 5C, D**). According to the SHAP Force Plot, in one control sample, the hub genes exhibit a negative contribution to the occurrence of OSA (**Figure 5E**). These results suggest that BTG2 might act as a protective gene, with low expression increasing risk, while UCHL1 and PRCP may serve as risk genes, with higher expression correlating with an increased risk of OSA.

### 3.6 Correlation analysis of immunity

**Figure 6:**
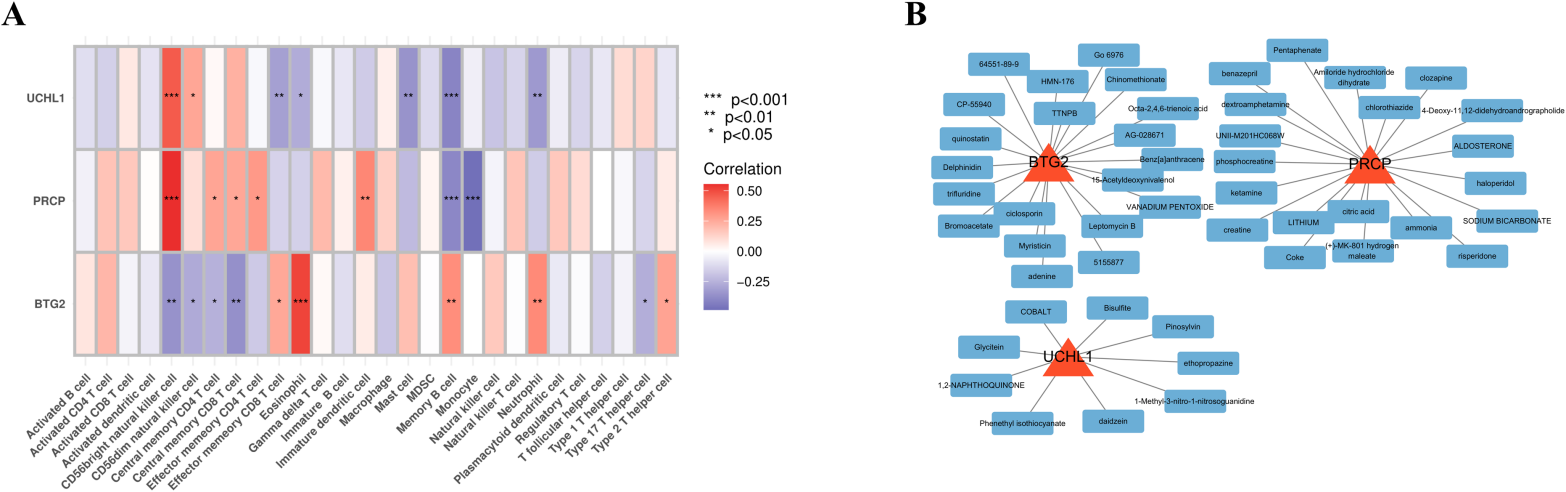
Immune association and potential targeted drugs of feature genes. **(A)** Heatmap depicting the correlations between immune cell infiltration and feature gene expression. **(B)** Network of drug-gene interactions, visualized using Cytoscape, showing potential therapeutic drugs targeting the feature genes.

The ssGSEA analysis was employed to calculate the immune cell infiltration scores, which were subsequently correlated with the expression levels of the hub genes. UCHL1 and PRCP demonstrated a strong positive correlation with CD56 bright natural killer cells and a significant negative correlation with Memory B cells. In contrast, BTG2 exhibited an inverse correlation pattern. A heatmap was generated to visualize the correlations between the other immune cells and the feature genes, providing a comprehensive overview of the immune landscape associated with these feature genes (**Figure 6A**).

### 3.7 Drug enrichment analysis

Using the gene IDs PRCP, UCHL1, and BTG2 as input in an online platform, 69 potential drugs were identified (p-value < 0.05). Among these, compounds such as Pentaphenate and Delphinidin exhibited significant associations with specific genes like PRCP and BTG2. Functional enrichment analysis highlighted their fold enrichment, z-scores, and adjusted p-values, suggesting that these compounds may exert critical biological effects on the related genes. Moreover, they could play regulatory roles in specific biological processes, providing insights into potential therapeutic applications. The network diagram illustrates the connections between identified drugs and their corresponding feature genes (**Figure 6B**). Each node represents a drug or a gene, with a maximum of 20 drugs displayed per gene.

### 3.8 Vivo and vitro experimental validation on multiple independent cohorts

**Figure 7:**
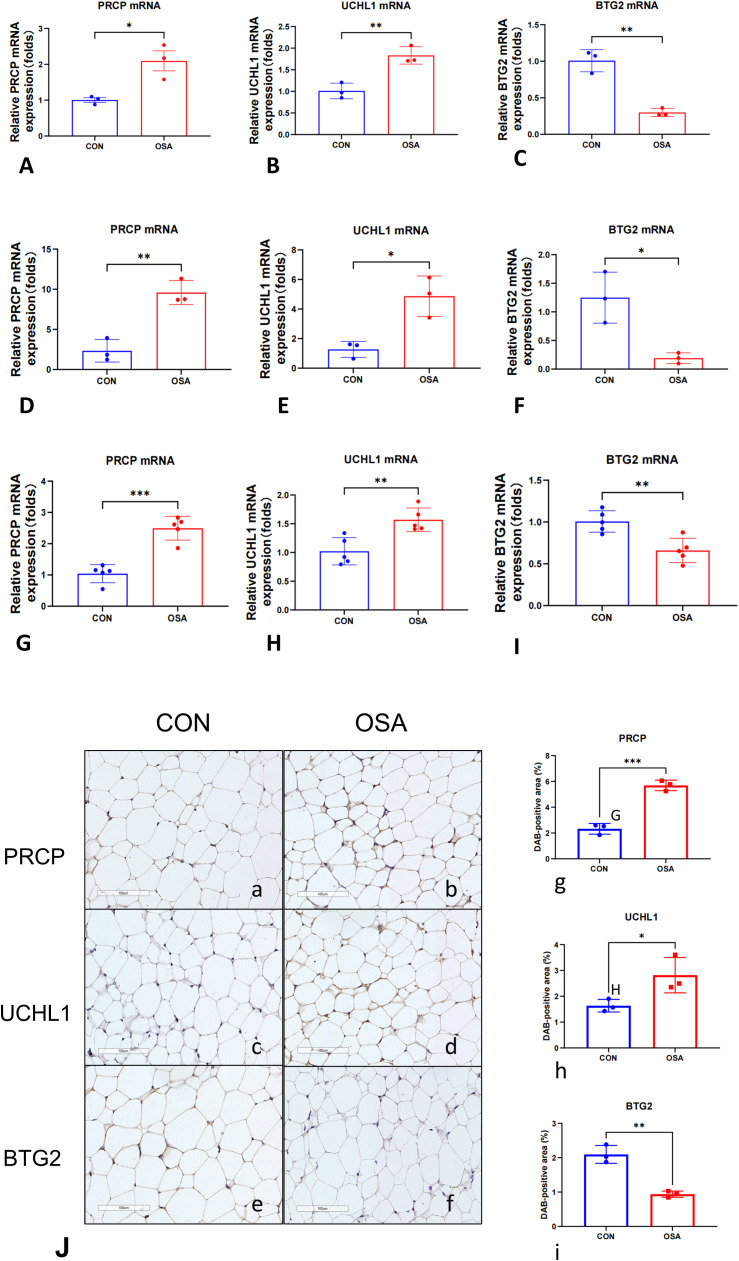
Intermittent hypoxia induces a conserved cellular stress signature across adipocyte models *in vitro* and *in vivo*. Relative mRNA expression of PRCP, UCHL1, and BTG2 was measured by qRT-PCR. **(A–C)** Differentiated 3T3-L1 murine adipocytes (n = 3 per group). **(D–F)** SW872 human adipocytes (n = 3 per group). Cells were exposed to 24 hours of normoxia or intermittent hypoxia (IH). **(G–I)** Epididymal white adipose tissue (eWAT) from mice exposed to 4 weeks of normoxia (Control, n = 5) or chronic intermittent hypoxia (CIH, n = 5). Gene expression was normalized to Actb. Data are presented as mean ± SEM. Statistical significance was assessed by unpaired, two-tailed Student’s t-test on ΔCt values. (**J**, a, b) Representative IHC images of PRCP, (**J**, c, d) UCHL1, (**J**, e, f) BTG2. Brown DAB precipitate indicates positive staining; nuclei are counterstained blue (scale bar = 100 μm). (**J**, g–i) Quantification of DAB-positive area (%). Each dot represents one independent sample; bars denote mean ± SD. *p < 0.05, **p < 0.01, ***p < 0.001.

To determine whether OSA induces transcriptional alterations in stress and metabolism-related genes, we performed qRT-PCR in multiple independent cohorts. We focused on PRCP, UCHL1, and BTG2, given their previously reported roles in proteolytic regulation, protein homeostasis, and cell cycle control. As shown in **Figures 7A–C** (Differentiated 3T3-L1 murine adipocytes), PRCP and UCHL1 mRNA levels were significantly elevated in the OSA group compared with controls, whereas BTG2 mRNA expression was markedly reduced. These findings were consistently reproduced in independent experimental sets (**Figures 7D–F**, SW872 human adipocytes; **Figures 7G–I**, eWAT), where PRCP upregulation was highly significant (**Figures 7D, G**), UCHL1 expression was robustly increased (**Figures 7E, H**), and BTG2 levels were consistently downregulated across all comparisons (**Figures 7F, I**). Notably, the concordant results across independent replicates underscore the stability and reproducibility of these transcriptional changes. Collectively, these data indicate that OSA is associated with a reproducible transcriptional signature characterized by increased PRCP and UCHL1 expression and decreased BTG2 expression. IHC: Representative micrographs (**Figures 7J**a–f) and semi-quantitative analysis of DAB-positive area (%) (**Figures 7J**g–i) revealed group-dependent differences (n = 3 per group). Compared with the control (CON) group, OSA samples exhibited significantly higher UCHL1 and PRCP expression and markedly lower BTG2 levels.

## 4 Discussion

This study elucidates the potential pathogenesis of OSA through adipose tissue transcriptomics, revealing PRCP, UCHL1, and BTG2 as exosome-associated hub genes that orchestrate metabolic-immune dysregulation. By synergizing cross-platform data integration (GSE135917/GSE38792), machine learning-driven biomarker discovery (XGBoost AUC = 0.968), and single-sample immune deconvolution, we reveal an unprecedented “exosome-immune” axis in OSA pathophysiology. Our robust feature selection pipeline—incorporating LASSO regularization (λ. min) and random forest permutation importance—convergently identified PRCP (prolyl carboxypeptidase), UCHL1 (Ubiquitin C-Terminal Hydrolase L1), and BTG2 (B-cell translocation gene 2) as key non-redundant classifiers, validated through SHAP interpretability to dissect nonlinear gene-disease interactions (SHAP value for PRCP: 0.334). These results not only demonstrate the diagnostic potential of these biomarkers but also highlight the utility of interpretable machine learning techniques in elucidating complex biological relationships (32).

Prolyl carboxypeptidase (PRCP), a serine protease, exerts regulatory effects across multiple endocrine axes including the renin-angiotensin system (RAS), kallikrein-kinin system (KKS), and pro-opiomelanocortin (POMC) (33). Study had demonstrated that PRCP plays a crucial role in the onset and progression of obesity, regulating the balance between energy intake and expenditure through an α-MSH1-mediated mechanism (34). The coexistence of obesity and OSA is commonly observed, with a bidirectional relationship between the two conditions (35). UCHL1, a key member of the deubiquitinating enzyme family, influences cell proliferation, differentiation, and damage by modulating both ubiquitination and non-ubiquitination pathways (36, 37). Notably, HIF-1α has been identified as a potential target interacting with UCHL1, and under hypoxic conditions, UCHL1 may regulate the nuclear translocation of HIF-1α, influencing its role in cellular responses to low oxygen levels (38). In OSA patients, IH activates HIF-1α, which in turn triggers systemic inflammation and disrupts hepatic lipid metabolism (39–41). BTG2, a member of the ERBB2 (BTG/TOB) family, functions as a B-cell transducer and regulator (42). Research has shown that Btg2 expression is elevated in the subcutaneous adipose tissue of obese mice on a high-fat diet, highlighting its involvement in lipid metabolism during obesity and metabolic disorders (43). Specifically, Btg2 reduces interleukin-6 expression by inhibiting the Stat3 signaling pathway, which plays a pivotal role in adipocyte differentiation (44, 45).

Moreover, our immune correlation analysis using ssGSEA revealed significant associations between the expression of the hub genes and various immune cell populations. Specifically, UCHL1 and PRCP showed strong positive correlations with CD56 bright natural killer cells and significant negative correlations with Memory B cells, whereas BTG2 exhibited an opposing pattern. During the differentiation process of monocytes into M1 macrophages, a significant upregulation of PRCP activity is observed (46). Studies have shown that human blood-derived alveolar macrophages exhibit higher PRCP activity (47, 48). Given that M1 macrophages are defined as pro-inflammatory macrophages, this suggests that PRCP plays a key role in the inflammatory response mechanism (46). Additionally, PRCP is also highly expressed in human neutrophils (49). UCHL1 primarily promotes the polarization of M1 macrophages by regulating the PI3K/AKT signaling pathway (50). It can also modulate the inflammatory response in lipopolysaccharide (LPS)-activated macrophages through MAPK and NF-κB signaling pathways (51). BTG2 mainly by controlling cell proliferation and activation processes to maintain T cell quiescence (42). Moreover, the protein complex formed by BTG2 and PRMT1 can effectively counteract the proliferation activity of pre-B cells, thus promoting the development of B cells (52). These findings provide solid evidence supporting the theory that exosome-related genes are involved in immune regulation, fully revealing their key positions and mechanisms of action within the immune regulation network.

In addition, drug enrichment analysis using the DSigDB platform identified several candidate compounds, such as Pentaphenate and Delphinidin, that significantly interact with the hub genes. Previous study had shown that PRCP, through its involvement in the pro-opiomelanocortin (POMC) system, makes it a highly promising target in the treatment of obesity and related diseases (34, 53). *In vitro* and *in vivo* experiments indicate that myricetin may influence the lipid metabolic process in the adipose tissue of obese mice by regulating the expression levels of miR-222 and its target gene BTG2 (54). These potential therapeutic agents may modulate exosome-mediated signaling and immune responses, offering promising avenues for targeted intervention in OSA.

While our machine learning approaches provides novel insights, limitations warrant consideration. First, the analyses were based exclusively on adipose tissue transcriptomic data, which may not fully reflect the systemic pathophysiology of OSA involving airway, liver, and circulating immune cells. Second, the relatively small sample size (n=60) may limit the generalizability of the results, underscoring the need for validation in larger, multi-center cohorts. Finally, the DSigDB-based drug predictions require experimental confirmation of target engagement and efficacy.

Based on previous research, we have developed an innovative hypothesis: the “Exosome-Immune Axis in the Pathogenesis of OSA.” During the progression of OSA, IH likely activates the HIF-1α signaling pathway in adipose tissue, leading to the release of pathological exosomes. These exosomes carry key regulatory molecules such as PRCP, UCHL1, and BTG2, initiating a vicious cycle of “hypoxia-exosome-immune metabolic disorder.” In terms of specific mechanisms, PRCP in the exosomes may enhance the differentiation of M1 macrophages and disrupt the normal metabolism of α-MSH, thereby triggering a systemic inflammatory response. UCHL1 may regulate the nuclear translocation of HIF-1α and activate the PI3K/AKT signaling pathway, further exacerbating M1 macrophage polarization and suppressing NK cell activity. BTG2 primarily affects lipid metabolism via the STAT3 signaling pathway and, through the BTG2-PRMT1 protein complex, promotes the differentiation and maturation of B cells. This model comprehensively integrates interactions involving “hypoxia-exosome”-mediated signaling, immune cell functional remodeling, and metabolic disruption, offering a promising new research direction for a deeper understanding of the systemic pathological mechanisms of OSA. Finally, the proposed “Hypoxia-Exosome-Immune Axis” represents a hypothesis derived from bioinformatics associations rather than demonstrated causal relationships, and its mechanistic details await functional validation.

In summary, this study is the first to identify PRCP, UCHL1, and BTG2 as exosome-based biomarkers associated with the diagnosis of OSA. These biomarkers are closely linked to immune-metabolic imbalance in the body. The findings not only uncover key molecular nodes involved in immune-metabolic disruption in the pathogenesis of OSA but also provide potential theoretical support and direction for the development of targeted therapeutic strategies based on the OSA exosome-immune axis.

## 5 Conclusion

This study identifies PRCP, UCHL1, and BTG2 as key exosome-related biomarkers in OSA that contribute to immune–metabolic dysregulation. By integrating transcriptomic data, machine learning, immune profiling, and *in vivo* and *in vitro* validations across multiple independent cohorts, we reveal an “exosome–immune” axis underlying OSA pathophysiology.

## Data Availability

Publicly available datasets were analyzed in this study. This data can be found here: The datasets generated and analyzed during the current study are available from the corresponding author on reasonable request.
