# Supplementary material for: Decoding the hypoxia-exosome-immune triad in OSA: PRCP/UCHL1/BTG2-driven metabolic dysregulation revealed by interpretable machine learning
Source: Front Immunol. 2025 Oct 27;16:1587522. doi: 10.3389/fimmu.2025.1587522 (PMC12597927; doi:10.3389/fimmu.2025.1587522)
Supplement: Supplementary file 1 [file DataSheet1.zip › Supplementary method and table.docx]

**Supplementary method and table**

**Cell Lines and Culture Conditions**

Human SW872 liposarcoma cells (SNL-524) were maintained in L-15 medium (Leibovitz) supplemented with 10% fetal bovine serum (FBS) and 1% penicillin-streptomycin. Cells were cultured at 37°C in a humidified incubator under a 100% air atmosphere. Murine 3T3-L1 preadipocytes (SNL-022) were maintained in Dulbecco's Modified Eagle's Medium (DMEM) supplemented with 10% calf serum (CS) and 1% penicillin-streptomycin at 37°C in a 5% CO₂ incubator.

**Adipogenic Differentiation of 3T3-L1 Cells**

3T3-L1 preadipocytes were seeded onto culture plates pre-coated with Gelatin Coating Solution. Adipogenesis was initiated at 95-100% confluence using a commercial 3T3-L1 Adipogenic Induction and Differentiation Kit (Cat# G4118, Servicebio). Per the manufacturer’s protocol, cells were treated sequentially with Induction Medium A for 48 hours, followed by Induction Medium B for 48 hours. Cells were subsequently maintained in Induction Medium B, with media changes every two days, for 7-14 days until the formation of mature, lipid-laden adipocytes was observed [1].

**Animal Model**

All animal experiments were performed in accordance with protocols approved by the Institutional Animal Care and Use Committee. Male C57BL/6J mice (8 weeks of age) were randomly assigned to normoxia (Control, n=5) or chronic intermittent hypoxia (CIH, n=5) groups for 4 weeks. Animals were housed under a 12-h light/dark cycle with ad libitum access to food and water [2].

**Intermittent Hypoxia (IH) Exposure**

For in vitro studies, differentiated 3T3-L1 or SW872 cells (n=3 per group) were subjected to 24 hours of intermittent hypoxia in a multi-gas incubator（Thermo）, with O₂ levels cycled between 1% (30 min) and 21% (30 min). For in vivo studies, mice were exposed to CIH during the 12-hour light cycle, wherein the fractional inspired O₂ (FiO₂) was cycled between 21% and 10% every 2 minutes. Control cells and animals were maintained under their respective standard normoxic conditions in parallel [3,4].

**Quantitative Real-Time PCR (qRT-PCR)**

Total RNA was isolated from cell lysates or snap-frozen epididymal white adipose tissue (eWAT)[5] using the SteadyPure Universal RNA Extraction Kit (Cat# AG21017, Accurate Biotechnology). The protocol included an on-column gDNA removal step with the provided gDNA Eraser Mini Columns. RNA concentration and purity were determined spectrophotometrically [6].

First-strand cDNA was synthesized from 1 µg of total RNA using the Evo M-MLV RT Kit for qPCR (Cat# AG11707, Accurate Biotechnology). The reverse transcription was primed with a mixture of Oligo(dT)18 and Random 6-mers. The reaction was incubated at 37°C for 15 min, followed by enzyme inactivation at 85°C for 5 s.

Gene expression was quantified by qRT-PCR on a CFX Connect Real-Time PCR Detection System (Biorad, USA) using the SYBR Green Premix Pro Taq HS qPCR Kit (Cat# AG11701, Accurate Biotechnology). The thermal cycling protocol was initiated with a 30 s denaturation at 95°C, followed by 40 cycles of 95°C for 5 s and 60°C for 30 s. Product specificity was confirmed by melt curve analysis. Relative transcript abundance was calculated using the 2^(-ΔΔCT) equation, with normalization to the housekeeping gene GAPDH. All reactions were performed in triplicate [7].

**Immunohistochemistry (IHC)**

FFPE iWAT sections (4–5 μm) were deparaffinized, rehydrated, and antigen-retrieved (10 mM citrate pH 6.0 or Tris–EDTA pH 9.0, as optimized). After 3% H₂O₂ (10 min) and serum/1% BSA (30 min) blocking, sections were incubated overnight at 4 °C with rabbit primaries PRCP (Proteintech 15995-1-AP, 1:200), BTG2 (Proteintech 22339-1-AP, 1:200), or UCHL1/PGP9.5 (Proteintech 84154-4-RR, clone 241107B2, 1:200), followed by HRP-polymer/DAB and hematoxylin; positive-tissue and isotype/no-primary controls were included [8].

Images were acquired under identical settings. Per animal, ≥5 non-overlapping 400× fields per section were analyzed across 2–3 non-adjacent sections (≥50 μm). The endpoint was area% (percentage positive area; or IOD/area) measured in ImageJ, averaging field values to one value per sample.

**Statistical Analysis.**

Data are expressed as mean ± SEM. For qPCR, per-sample ΔCt values were tested;2^−ΔΔCt is reported for presentation [7] in accordance with MIQE [6]. For IHC, the prespecified endpoint was area% (percentage positive area) averaged per sample across predefined fields. Two groups were compared with unpaired two-tailed t-tests; ≥3 groups with one-way ANOVA and appropriate post-hoc tests. Assumptions of normality/homoscedasticity were checked, with non-parametric alternatives used as required. P < 0.05 was considered significant (GraphPad Prism 9).

**Supplementary table.** The primer sets.

| Gene ID | Primer | Sequence |
| --- | --- | --- |
| 5547 | **Human-PRCP-F** | GAAAAGCGGTCCACATTGTTC |
|  | **Human-PRCP-R** | ATGGGCTGCATAAGTGAAGGG |
| 7345 | **Human-UCHL1-F** | CCTGTGGCACAATCGGACTTA |
|  | **Human-UCHL1-R** | CATCTACCCGACATTGGCCTT |
| 7832 | **Human-BTG2-F** | CCTGTGGGTGGACCCCTAT |
|  | **Human-BTG2-R** | GGCCTCCTCGTACAAGACG |
| 2597 | **Human-GAPDH-F** | CTGGGCTACACTGAGCACC |
|  | **Human-GAPDH-R** | AAGTGGTCGTTGAGGGCAATG |
| 72461 | **Mouse-Prcp-F** | TAGTCGCTGATAAACACTGGCA |
|  | **Mouse-Prcp-R** | CCCACATGAACCCCGTATTATTG |
| 22223 | **Mouse-Uchl1-F** | AGGGACAGGAAGTTAGCCCTA |
|  | **Mouse-Uchl1-R** | AGCTTCTCCGTTTCAGACAGA |
| 12227 | **Mouse-Btg2-F** | GGCTATCGCTGTATCCGTATCA |
|  | **Mouse-Btg2-R** | TGCGGTAAGACACTTCATAGGG |
| 14433 | **Mouse-Gapdh-F** | AGGTCGGTGTGAACGGATTTG |
|  | **Mouse-Gapdh-R** | TGTAGACCATGTAGTTGAGGTCA |

**References**

[1] Zebisch K, Voigt V, Wabitsch M, Brandsch M. Protocol for effective differentiation of 3T3-L1 cells to adipocytes. Anal Biochem. 2012;425(1):88-90. doi:10.1016/j.ab.2012.03.005

[2] National Research Council (US) Committee for the Update of the Guide for the Care and Use of Laboratory Animals. Guide for the Care and Use of Laboratory Animals. 8th ed. Washington (DC): National Academies Press (US); 2011.

[3]Ryan S. Adipose tissue inflammation by intermittent hypoxia: mechanistic link between obstructive sleep apnoea and metabolic dysfunction. J Physiol. 2017;595(8):2423-2430. doi:10.1113/JP273312

[4] Trzepizur W, Gaceb A, Arnaud C, et al. Vascular and hepatic impact of short-term intermittent hypoxia in a mouse model of metabolic syndrome. PLoS One. 2015;10(5):e0124637. Published 2015 May 18. doi:10.1371/journal.pone.0124637

[5] Weisberg SP, McCann D, Desai M, Rosenbaum M, Leibel RL, Ferrante AW Jr. Obesity is associated with macrophage accumulation in adipose tissue. J Clin Invest. 2003;112(12):1796-1808. doi:10.1172/JCI19246

[6] Bustin SA, Benes V, Garson JA, et al. The MIQE guidelines: minimum information for publication of quantitative real-time PCR experiments. Clin Chem. 2009;55(4):611-622. doi:10.1373/clinchem.2008.112797.

[7] Livak KJ, Schmittgen TD. Analysis of relative gene expression data using real-time quantitative PCR and the 2^−ΔΔCT method. Methods. 2001;25(4):402-408. doi:10.1006/meth.2001.1262.

[8]Murano I, Barbatelli G, Parisani V, et al. Dead adipocytes, detected as crown-like structures, are prevalent in visceral fat depots of genetically obese mice. J Lipid Res. 2008;49(7):1562-1568. doi:10.1194/jlr.M800019-JLR200.
